# Assessing phenotypic virulence of *Salmonella enterica* across serovars and sources

**DOI:** 10.3389/fmicb.2023.1184387

**Published:** 2023-06-06

**Authors:** Sara Petrin, Lucas Wijnands, Elisa Benincà, Lapo Mughini-Gras, Ellen H. M. Delfgou-van Asch, Laura Villa, Massimiliano Orsini, Carmen Losasso, John E. Olsen, Lisa Barco

**Affiliations:** ^1^Microbial Ecology and Microrganisms Genomics Laboratory, Istituto Zooprofilattico Sperimentale delle Venezie, Legnaro, Padova, Italy; ^2^Department of Veterinary and Animal Sciences, Faculty of Health and Medical Sciences, University of Copenhagen, Frederiksberg C, Denmark; ^3^Centre for Infectious Disease Control (CIb), National Institute for Public Health and the Environment (RIVM), Bilthoven, Netherlands; ^4^Institute for Risk Assessment Sciences (IRAS), Utrecht University, Utrecht, Netherlands; ^5^Department of Infectious Diseases, Istituto Superiore di Sanità, Rome, Italy; ^6^WHOA and National Reference Laboratory for Salmonellosis, Istituto Zooprofilattico Sperimentale delle Venezie, Legnaro, Padova, Italy

**Keywords:** *Salmonella enterica*, whole genome sequencing, phenotypic virulence, Bayesian approach, gastrointestinal tract model system, probability of infection, virulence genes

## Abstract

**Introduction:**

Whole genome sequencing (WGS) is increasingly used for characterizing foodborne pathogens and it has become a standard typing technique for surveillance and research purposes. WGS data can help assessing microbial risks and defining risk mitigating strategies for foodborne pathogens, including *Salmonella enterica*.

**Methods:**

To test the hypothesis that (combinations of) different genes can predict the probability of infection [P(inf)] given exposure to a certain pathogen strain, we determined P(inf) based on invasion potential of 87 *S. enterica* strains belonging to 15 serovars isolated from animals, foodstuffs and human patients, in an *in vitro* gastrointestinal tract (GIT) model system. These genomes were sequenced with WGS and screened for genes potentially involved in virulence. A random forest (RF) model was applied to assess whether P(inf) of a strain could be predicted based on the presence/absence of those genes. Moreover, the association between P(inf) and biofilm formation in different experimental conditions was assessed.

**Results and Discussion:**

P(inf) values ranged from 6.7E-05 to 5.2E-01, showing variability both among and within serovars. P(inf) values also varied between isolation sources, but no unambiguous pattern was observed in the tested serovars. Interestingly, serovars causing the highest number of human infections did not show better ability to invade cells in the GIT model system, with strains belonging to other serovars displaying even higher infectivity. The RF model did not identify any virulence factor as significant P(inf) predictors. Significant associations of P(inf) with biofilm formation were found in all the different conditions for a limited number of serovars, indicating that the two phenotypes are governed by different mechanisms and that the ability to form biofilm does not correlate with the ability to invade epithelial cells. Other omics techniques therefore seem more promising as alternatives to identify genes associated with P(inf), and different hypotheses, such as gene expression rather than presence/absence, could be tested to explain phenotypic virulence [P(inf)].

## 1. Introduction

Non-typhoidal *Salmonella enterica* (NTS) is the second most common causative agent among reported human zoonotic infections in the European Union (EU) (EFSA and ECDC, 2021a) and one of the major challenges facing food safety and public health nowadays. Different serovars have been implicated in foodborne outbreaks and human cases of salmonellosis, but three of them, namely *Salmonella* (hereafter *S.*) Enteritidis, *S. Typhimurium* and the monophasic variant of *S. Typhimurium* (MVST), account for over 70% of the confirmed human cases in Europe (EFSA and ECDC, 2021a,b). Other *Salmonella* serovars are also commonly implicated in human infections, but their occurrence among clinical cases is lower. For instance, *S. Infantis*, *S*. Newport and *S*. Derby altogether accounted for only 4.4% of the confirmed human cases in 2019 (EFSA and ECDC, 2021b).

NTS is mainly spread from farm animals to humans through food sources. *S. Typhimurium* and MVST are primarily associated with pigs and chickens, *S*. Derby with pigs and turkeys, and *S. Enteritidis* and *S. Infantis* with layers and chickens, respectively (EFSA and ECDC, 2021a,b). Foodstuffs are important sources for human salmonellosis, as highlighted by the number of foodborne outbreaks in which foods have been strongly implicated in recent years (EFSA and ECDC, 2017; EFSA and ECDC, 2021a,b; Chanamè-Pinedo et al., 2022b). To lower the prevalence of *Salmonella* along the food chain, from farm to fork, control measures have been implemented in the EU. In particular, national control programs to reduce the prevalence of *Salmonella* in poultry farms have been established according to EU Regulation (EC) No 2160/2003. Consequently, serovars that are relevant to public health, namely *S. Enteritidis*, *S. Typhimurium* and MVST, are considered target *Salmonella* serovars in poultry flocks. On the contrary, only few countries have implemented control programs for *Salmonella* in pigs, and these programs are not harmonized across the EU (Bonardi et al., 2021; Correia-Gomes et al., 2021). In addition, EU Regulation (EC) No 2073/2005, sets the microbiological criteria for *Salmonella* in foodstuffs, determining food safety and process hygiene criteria for both poultry and pig products, as well as other food matrices.

Despite the implementation of control plans in the poultry production chain, no significant decrease in human salmonellosis has been observed since 2012 (Koutsoumanis et al., 2019b; Chanamè-Pinedo et al., 2022a), challenging the current choice of the identified target *Salmonella* serovars. Indeed, a higher impact on public health might be expected if the selection of target serovars is amended according to their occurrence and epidemiology in each European country. Moreover, it has been proposed to select serovars considering additional criteria than those currently used, including antimicrobial resistance and virulence characteristics [Annex III from the Commission Regulation (EU) No 2160/2003; Koutsoumanis et al., 2019a; Leati et al., 2021]. The focus on serovar without consideration of the actual pathogenicity potential of the single strain for animals and humans might overlook highly pathogenic *Salmonella* strains, which do not necessarily belong to the target serovars, and such strains could spread as emerging clones, becoming potential causes of new outbreaks.

The success of *Salmonella* as a pathogen results from the ability to cause acute intestinal inflammation in many host species, including humans, differently from close relatives such as *E. coli*. The invasion of epithelial cells evokes an acute inflammatory reaction in the intestinal mucosa that ultimately involves neutrophils, which, during infection, generate reactive oxygen species (ROS) in the intestinal lumen. As a consequence, ROS produced during inflammation oxidize thiosulfate, a metabolite generated from the oxidation of hydrogen sulphide produced by the fermenting microbiota, to tetrathionate. *Salmonella* can use tetrathionate as a terminal electron acceptor in tetrathionate respiration (Hensel et al., 1999), and the induced acute intestinal inflammation provides a significant growth advantage over competing microbes, which rely only on fermentation to obtain energy for growth (Bäumler et al., 2011; Rivera-Chávez and Bäumler, 2015).

The evolution of *Salmonella* as a pathogen occurred over time also through the acquisition of genetic material by horizontal gene transfer and genome erosion through pseudogenes formation, and led to host adaptation of a number of *Salmonella* serovars and variability in terms of infection outcomes (Uzzau et al., 2000; Matthews et al., 2015; Parisi et al., 2018). Moreover, the ability of *Salmonella* to colonize and cause disease in different hosts could also depend on the allelic variations within a collection of specific virulence genes or effector proteins (Yue and Schifferli, 2014), and different studies have demonstrated how allelic variations affected *Salmonella* pathogenesis (Hopkins and Threlfall, 2004; Thornbrough and Worley, 2012; Yue et al., 2015; Rakov et al., 2019) and contributed to bacterial adaptation to preferential hosts (De Masi et al., 2017).

The potential of whole genome sequencing (WGS) in the identification and characterization of foodborne pathogens has been widely recognized to the point that it is now becoming a standard surveillance technique for epidemiological purposes (Koutsoumanis et al., 2019a), and the amount of data generated by WGS could potentially be used in defining microbial risk and to set mitigation strategies aimed at reducing the human cases caused by zoonotic *Salmonella*.

Building upon the hypothesis that different genes can predict the probability of infection (P(inf)), given exposure to a certain strain and thus help define the public health relevance of such strain, here we determined P(inf), as a proxy of infectivity, based on the invasion potential of 87 *Salmonella* strains belonging to 15 different serovars using an *in vitro* gastrointestinal tract (GIT) model system (Pielaat et al., 2016; Wijnands et al., 2017). We further applied a random forest model to assess whether the P(inf) of a strain can be predicted as a function of the presence/absence of a set of genes related to virulence or as a function of its ability to form biofilm.

## 2. Materials and methods

### 2.1. Bacterial strains

Eighty-seven *S. enterica* strains, belonging to the selection of isolates already described in Petrin et al. (2022), were included in this study. Briefly, the strains were isolated in Italy between 2009 and 2019 and belonged to the collection of the Italian National Reference Laboratory for Salmonellosis, Istituto Zooprofilattico Sperimentale delle Venezie (IZSVe) and the Istituto Superiore di Sanità (ISS). The strains belonged to 15 different serovars, representing both frequently occurring serovars in relation to human salmonellosis cases in Italy and other European countries (EFSA and ECDC, 2021a) [i.e., *S. Enteritidis*, *S. Typhimurium* and monophasic variant of *S. Typhimurium* (MVST)], and more rarely occurring serovars (i.e., *S*. Derby, *S*. Dublin, *S*. Hadar, *S. Infantis*, *S*. Kentucky, *S*. Livingstone, *S*. Mbandaka, *S*. Montevideo, *S*. Newport, *S*. Rissen, *S*. Senftenberg, and *S*. Thompson). Strains were isolated from animal, food and human sources details regarding the selected strains are reported in Supplementary Table S1 (Sheet 1, Bacterial strains). The strains were maintained at −80°C on cryobank beads (Microbank, Pro-Lab Diagnostics) until use. Single bacteria colonies were cultured in BHI-broth overnight at 37°C for testing in a simulated gastro-intestinal tract (GIT) system assay, in which a *S*. Typhimurium isolate (STM 3283) was included as control, as described in detail elsewhere (Wijnands et al., 2017).

### 2.2. Genome sequences for *Salmonella* pangenome

A collection of 759 *Salmonella* genome sequences, including those of the 87 strains used in the GIT assay, were selected to build the *Salmonella* pangenome for this study. Of the genomic sequences, 370 genomes were newly sequenced as described in Section 2.3, while 389 genomes were downloaded from EnteroBase [Last access 03/06/2019 (Zhou et al., 2020)]^1^, among *Salmonella* genomic sequences using the same inclusion criteria in terms of serovar and source of isolation as for the GIT strains. The complete dataset is described in Supplementary Table S1 (Sheet 2, Genome sequences).

### 2.3. Whole genome sequencing

The *Salmonella* isolates were sequenced starting from pure culture on tryptose agar, grown overnight at 37°C. Genomic DNA (gDNA) was extracted using a commercial column-based kit (QIAamp DNA Mini, QIAGEN), and purified gDNA was quantified with a Qubit 3.0 Fluorometer (Life Technologies). Libraries for whole genome sequencing were prepared using the Nextera XT DNA sample preparation kit (Illumina) following the manufacturer’s instructions. High-throughput sequencing was performed with MiSeq Reagent kit v3, resulting in 251 bp long paired-end reads or NextSeq High Output kit v2.5, resulting in 151 bp long paired end reads. FastQC v0.11.2 (Andrews, 2010) was used to assess the sample quality, while Trimmomatic 0.32 (Bolger et al., 2014) was used to trim both quality and length, with the following options: removal of Nextera adapters sequences; cut bases off the start of the read, if below a quality score of 20; cut bases off the end of the read, if below a quality score of 20; sliding window trimming, clipping the read once the average quality within the window (4 bp) falls below 20; finally, drop the read if it is shorter than 100 bp (Mastrorilli et al., 2020). Subsequently, reads were *de novo* assembled using Spades 3.10.1 (Bankevich et al., 2012) with default parameters for Illumina reads, and the quality of assembly was assessed using QUAST 3.1 (Gurevich et al., 2013). Details about quality and metrics of the assemblies are reported in Supplementary Table S1.

### 2.4. Pangenome determination and database for genes prediction

*Salmonella* genomes were annotated using Prokka v1.14.5 with default parameters. The final pangenome was built using Roary v3.13.0, excluding singletons. At the same time, the 28,639 protein sequences collected in Virulence Factor database [VFDB, last access 08/01/2021, (Chen et al., 2005)] were downloaded and searched in the *Salmonella* pangenome. Protein sequences were preferred since proteins are usually the biological effectors and allow to not accounting for degenerate codons. The presence of a protein sequence in the pangenome was assessed with Diamond v0.9.17 (Buchfink et al., 2015), setting 90% coverage and 90% identity. Protein sequences that were found in all or none of the *Salmonella* genomes were excluded from the subsequent analyses. Moreover, a second subset of proteins (hereafter ‘informative sequences’), collecting only the sequences present in at least 10% and no more than 95% of the genomes, were used in the following analyses.

### 2.5. Gastro-intestinal tract system assay

**Figure 1:**
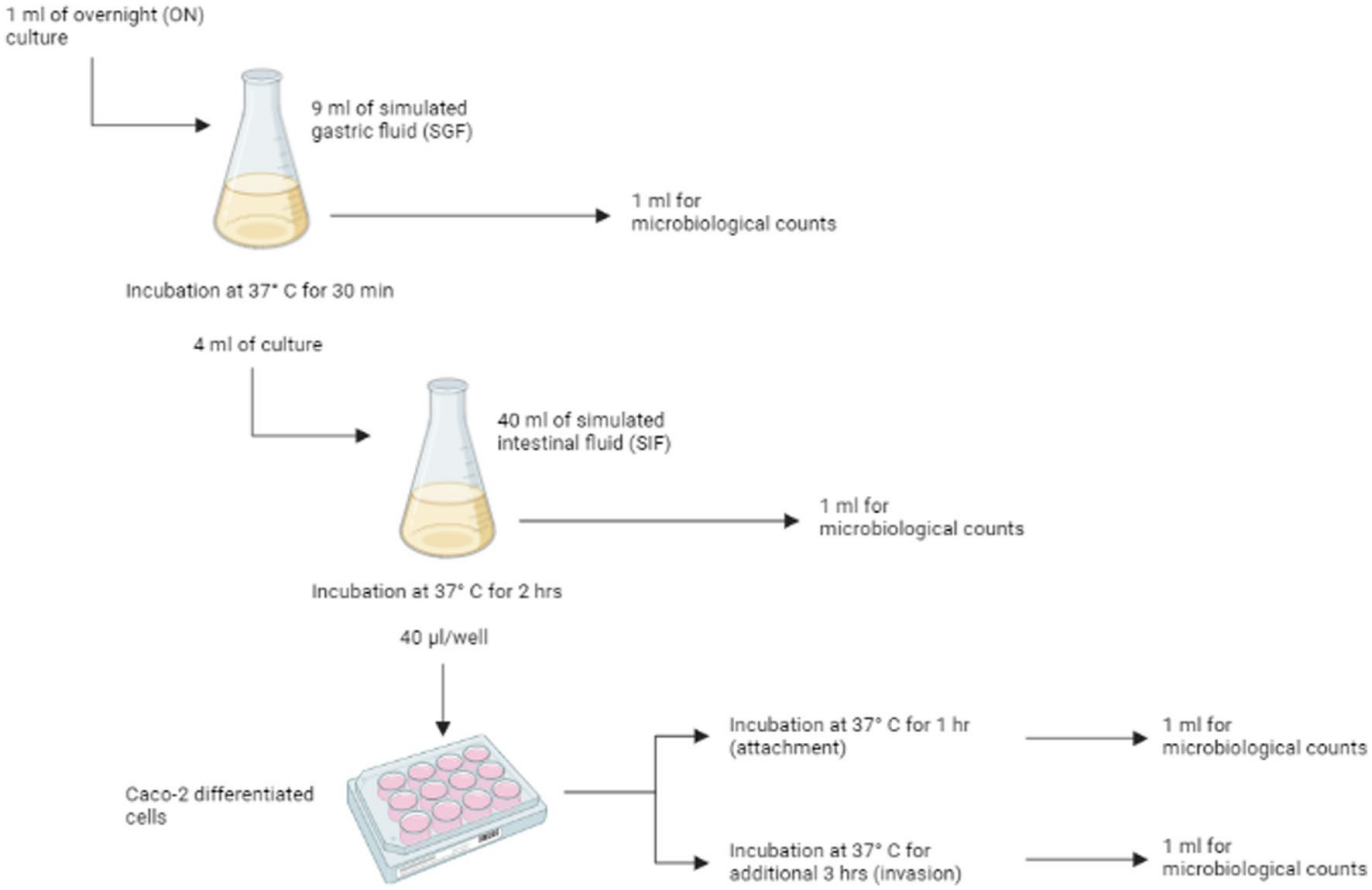
Schematic representation of the *in vitro* gastrointestinal tract (GIT) model system passages. For each of the different stages [ON, SGF, SIF, attachment (ATT) and invasion (INV)] replicate counts were prepared. Figure is created with BioRender.

The gastro-intestinal tract (GIT) model system developed by Wijnands et al. (2017) was used to quantify the *in-vitro* infectivity of *Salmonella* strains by estimating their *in vitro* probability of infection or P(inf). P(inf) is calculated as the ratio invasion (INV) to overnight (ON) bacterial concentrations (see below). The system is composed of four sequential stages through which *Salmonella* isolates are transferred without intermediate culturing: simulated gastric fluid (SGF), simulated intestinal fluid (SIF), attachment (ATT) and invasion (INV) (Figure 1). The composition and preparation of simulated gastric and intestinal fluids are reported in Supplementary material S1.

#### 2.5.1. Simulated gastrointestinal passages

One ml of overnight (ON) culture was added to 9 mL of SGF and incubated for 30 min at 37°C in a humidified atmosphere of 95% air – 5% CO_2_. After the incubation, 1 mL was used for enumeration of viable cells (see 2.5.3), while 4 mL were transferred to 40 mL of SIF and incubated for 2 h at 37°C, under microaerophilic conditions (6% O_2_), with shaking at 50 rpm. After that, surviving cells were used for the invasion/attachment assay (see 2.5.2) and enumerated.

#### 2.5.2. Attachment/invasion assay with Caco-2 cells

Caco-2 (HTB-37, ATCC) cells were cultured as described in Oliveira et al. (2011) and maintained in Dulbecco’s Modified Eagle’s Medium (DMEM, Gibco) with 10% heat-inactivated fetal bovine serum (FBS, Gibco), 1% non-essential amino acids (Gibco), 1% 100X glutamine (Gibco) and 0,1% gentamycin (50 mg/mL, Gibco) in 75 cm^2^ flasks (Corning Inc.). Caco-2 cells were grown to confluence at 37°C in a humidified atmosphere of 95% air – 5% CO_2_ and differentiated into cells simulating the small intestine epithelial cells by culturing the cells in monolayers, in 12-wells tissue culture plates (Corning Inc.). To achieve the differentiation, cells were seeded at a density of 1.6 × 10^5^ cell/ml and growth media was changed every 2 or 3 days. The cells completely differentiate in 14 days after being cultured (Pinto et al., 1983).

Caco-2 cells were washed three times with sterile phosphate buffered solution (PBS) before the assay, in order to remove traces of gentamycin antibiotic. After that, 1 mL of experimental culture medium (ECM, i.e., prewarmed DMEM without gentamycin and FBS) was added to each of the 12 wells. 40 μL of the cell mixture from the previous steps were inoculate in each well, and the plates were incubated at 37°C in a humidified atmosphere of 95% air – 5% CO_2_ for 1 h. After the incubation, the medium was discarded and monolayers were rinsed three times with sterile PBS, to remove non-attached or loosely attached bacteria. The cells were then used to determine both attached and invading bacteria and invading bacteria. For the enumeration of attached and invading bacteria, six wells of Caco-2 cells were lysed with 1% Triton-X100 (Merck) in PBS for 5 min at room temperature. The lysate from three wells was combined and named ATT1 and ATT2. The Caco-2 cells in the remaining six wells were treated with ECM supplemented with gentamycin (300 μg/mL) to inactivate attached bacteria (Berk, 2008) and incubated at 37°C in a humidified atmosphere of 95% air – 5% CO_2_. After 3 h, cells were washed three times with sterile PBS to remove residual antibiotic and lysed with 1% Triton-X100. To enumerate the invading bacteria, the lysate from three wells was combined and named INV1 and INV2.

#### 2.5.3. Enumeration of bacterial load

Single bacteria samples from overnight (ON) culture, SGF and SIF passages, and both the aliquots ATT1 and ATT2, and INV1 and INV2 were used to enumerate the bacterial load at each stage of the simulated gastrointestinal system. Appropriate 10-fold dilutions were prepared, and dilutions were plated on Tryptone soy agar (TSA) in duplicate. To estimate P(inf) (see below), duplicate counts for each appropriate dilution from the three first stages (ON, SGF and SIF), and duplicate counts for each appropriate dilution for ATT1, ATT2, INV1 and INV2 were considered.

### 2.6. Statistical analysis

#### 2.6.1. GIT system data analysis

The bacterial count data obtained from the GIT system were analyzed using a Bayesian model that is able to distinguish between experimental uncertainty and biological variability in the estimates of bacterial counts, as presented in details elsewhere (Wijnands et al., 2017; Kuijpers et al., 2019). Briefly, the measured bacterial counts in the different stages of the GIT system were assumed to follow a Poisson distribution, while the bacterial concentrations were assumed to follow a log-normal distribution and the log changes at any phase of the GIT were estimated by using a Markov chain Monte Carlo (MCMC) sampling scheme. The logarithm of the probability of infection, or P(inf), which provides an estimate of the *in vitro* infectivity of the tested isolate, was then defined as the sum of all log changes in bacterial concentrations throughout the GIT system passages, from the overnight culture (ON) to invasion of Caco-2 cells (INV). The outputs of the model resulted in a posterior distribution of P(inf) values for each *Salmonella* isolate tested with the GIT system. From the estimated distribution of P(inf), the mean value and the 95% confidence intervals were then calculated.

#### 2.6.2. Virulence difference testing

Multivariable generalized linear models (GLM) with Gamma error distribution and a log link function were used for statistical significance testing of the differences in P(inf) (dependent variable) among serovars and sources of isolation (independent variables). This GLM parameterization was chosen given the positively skewed P(inf) distribution. The same approach was used to test associations between P(inf) and biofilm formation ability under different experimental conditions, as studied by Petrin et al. (2022). Estimates were thus adjusted for differences among serovars and sources of isolations (covariates), and clustering of observation (replicates) at the isolate level using cluster-robust (Sandwich) variance estimators (Williams, 2000). Prior to GLM analysis, to limit the number of hypotheses tested and therefore minimize Type-I error, two-way ANOVA was used to screen whether there were significant differences among serovars (15 groups) and sources (3 groups) in each passage of the GIT system, i.e., survival to exposure to gastric and intestinal fluid, adhesion and invasion. Analyses were performed in STATA 17 (StataCorp, College Station, TX, USA). A *p* < 0.05 was considered statistically significant.

#### 2.6.3. Assessing the similarity of clusters based on virulence and genetic characteristics

To investigate the potential associations between the mean value of P(inf) and the presence or absence of specific virulence genes, an unsupervised cluster algorithm was used. A distance matrix was then built for mean P(inf) values based on the pairwise Euclidean distances between the mean P(inf) of each isolate, while the distance matrix for the gene presence/absence was built based on the Jaccard distance, defined as 1-Jaccard index. The Jaccard index was computed pairwise on each pair of isolates. From the calculated matrices, a hierarchical agglomerative algorithm based on the Ward method (Ward, 1963) was applied to build cluster trees. To quantify similarities between the two cluster trees, the *B_k_* statistics was used (Fowlkes and Mallows, 1983), which is defined as follows.

Considering two trees, each one with the same number of elements *n*, and partitioning each of them into *k* = 2, …, *n* − 1 sub-clusters, *B_k_* is then defined as:


(1) $$B_{k}=\frac{T_{k}}{\sqrt{P_{k}Q_{k}}};$$

with:


(2) $$T_{k}=\sum_{i=1}^{k}\sum_{j=1}^{k}s_{i,j}{\;}^{2}-n;$$

(3) $$P_{k}=\sum_{i=1}^{k}{\left(\sum_{j=1}^{k}s_{i,j}\;\right)}^{2}-n;$$

(4) $$Q_{k}=\sum_{j=1}^{k}{\left(\sum_{i=1}^{k}s_{i,j}\;\right)}^{2}-n;$$

and with *s_i,j_* quantifying the number of elements shared between the *i*th cluster of the first tree and the *j*th cluster of the second tree*. B*_k_ values range between 0 and 1, with 1 indicating complete correspondence and 0 indicating complete non-correspondence between the sub-clusters of the two trees. *B*_k_ has been computed by using the R package “Dendextend” (Galili, 2015) for all sub-clusters *k*.

#### 2.6.4. Predicting mean P(inf) from virulence genes and ability to produce biofilm

In order to assess whether the mean value of P(inf) could be predicted by the presence/absence of specific genes, a Random Forest model was applied by using the ‘randomForest’ package in R (RStudio Team, 2021). The same analysis was repeated using the informative sequences, ability to form biofilm and serovar as predictors (Supplementary material S1). The random forest models were applied in two ways: regression and classification mode. In the regression mode the variable of interest, P(inf), was considered as a continuous variable, while in the classification mode, P(inf) values were split in two categories: ‘low’ if P(inf) < median [P(inf)] and ‘high’ if P(inf) ≥ median [P(inf)]. The virulence genes from the presence/absence matrix, as well as informative sequences, ability to produce biofilm and serovar (Supplementary material S1), were used as model predictors after removing the non-informative records from the databases, i.e., those genes that were present or absent in all isolates. Proportion of explained variance (PEV) and out-of-bag (OOB) predictions were calculated after running the models with 10,000 trees.

## 3. Results

### 3.1. *In vitro* virulence

**Figure 2:**
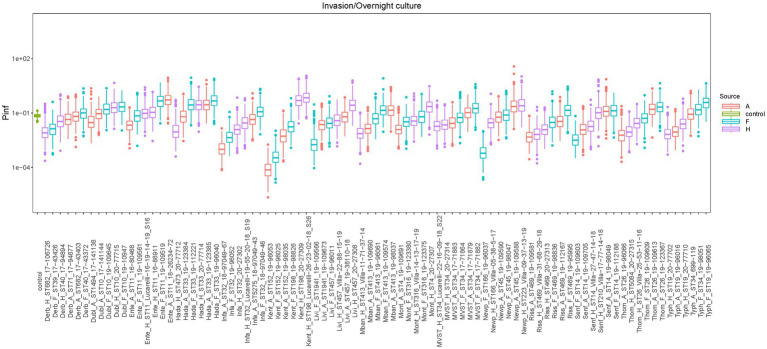
Boxplots of the estimated P(inf) values from the GIT system bacterial counts with the Bayesian model for each strain, sorted by serotype. Mean P(inf) is indicated with horizontal bold lines inside boxplots. Colors indicate the source of the isolate [red = animal sources (A in the strain name), light blue = food sources (F in the strain name), purple = human sources (H in the strain name), green = control strain (STM 3283 from Wijnands et al., 2017)]. Additional strains information can be found in Supplementary Table S2.

Box plots of the P(inf) values estimated with the Bayesian model for all tested isolates, sorted by serovars, are shown in Figure 2, while the measured P(inf) values from the GIT system are reported in Supplementary Table S2. A degree of variability existed between the tested strains, with a *S*. Kentucky isolate from an animal source having the lowest average P(inf), 6.7E-05, and an *S*. Kentucky strain from a human source having the highest average P(inf), 5.2E-01. At the serovar level, individual isolates displayed a wide range of P(inf) values, except for *S*. Hadar, for which most of the isolates (4 out of 6) showed similar *in vitro* virulence behavior, with P(inf) values being 51 to 55% lower than the P(inf) of the most virulent strain (Figure 2; Supplementary Table S2). Also *S.* Dublin showed small differences in P(inf) for the individual strains, with P(inf) values ranging from 1.93E-02 to 1.8E-01, corresponding to a variation of maximum 11% within *S*. Dublin strains, while *S.* Kentucky, *S.* Newport and *S. Typhimurium* displayed larger differences in P(inf).

Overall, there was no statistical difference in the estimated mean P(inf) values depending on the source (animal, food, human: value of *p* = 0.7159) or serovar (value of *p* = 0.3760).

Similar to the lack of statistical difference in P(inf) between serovars and sources, there were no statistically significant differences among serovars or sources in their invasiveness (value of *p*s = 0.1127 and 0.5192, respectively), intestinal survival, i.e., the fraction of bacteria surviving exposure to intestinal fluid over bacteria surviving exposure to gastric fluid (value of *p*s = 0.0879 and 0.0756, respectively) and gastric survival, i.e., the fraction of bacteria surviving exposure to gastric fluid over the original bacterial load introduced in the GIT system (value of *p*s = 0.7918 and 0.9585, respectively). The ANOVA test showed statistically significant differences in the attachment ability among the different serovars, i.e., the fraction of bacteria attaching to cells over bacteria surviving exposure to intestinal fluid (value of *p* = 0.0002). Thus, the Bonferroni-corrected comparisons in adhesiveness (post-hoc comparisons between serovars) showed that *S. Typhimurium* differed significantly from *S*. Livingstone (value of *p* = 0.004), MVST (value of *p* = 0.005), *S*. Mbandaka (value of *p* = 0.026), *S*. Rissen (value of *p* <0.001) and *S*. Senftenberg (value of *p* = 0.001). Conversely, differences among sources were not statistically significant (value of *p* = 0.8470).

### 3.2. Association of P(inf) with biofilm formation ability

The ability to form biofilm was significantly negatively associated with the size of P(inf) for *S. Infantis* grown in tryptic soy broth (TSB) (value of *p* = 0.000); *S*. Derby and *S*. Montevideo grown in TSB 4% NaCl, pH4.5 (value of *p*s = 0.045 and 0.000, respectively); and *S. Infantis* and *S*. Montevideo grown in TSB 10% NaCl, pH4.5 (value of *p*s = 0.030 and 0.000, respectively). In contrast, the ability to form biofilm was significantly and positively associated with the size of P(inf) for *S*. Hadar grown in TSB 4% NaCl, pH7 (*p*-value = 0.008) and *S. Enteritidis* and *S*. Hadar grown in TSB 10% NaCl, pH7 (*p*-values = 0.014 and 0.003, respectively). Significant results are reported in Table 1, extended results are reported in Supplementary Table S3.

**Table 1:** Significant results of the association test between P(inf) and biofilm formation ability in different experimental conditions for different serovars.

Experimental condition	Serovar	Beta^a^	Bonferroni-corrected 95% CI		p-value
TSB	*S. Infantis*	−6.581	−10.479	−2.682	0.000
TSB 4% NaCl pH 7	*S. Hadar*	7.844	1.312	14.376	0.008
TSB 10% NaCl pH 7	*S. Enteritidis*	12.481	1.656	23.306	0.014
	*S. Hadar*	11.405	2.548	20.262	0.003
TSB 4% NaCl pH 4.5	*S. Derby*	−15.909	−31.082	−0.736	0.045
	*S. Montevideo*	−9.378	−15.816	−2.940	0.000
TSB 10% NaCl pH 4.5	*S. Infantis*	−37.142	−72.260	−2.023	0.030
	*S. Montevideo*	−11.519	−19.258	−3.779	0.0

a Beta = coefficient of the GLM with a gamma error distribution and a log link function.

### 3.3. Assessing the similarity of clusters based on virulence genes and genetic characteristics

**Figure 3:**
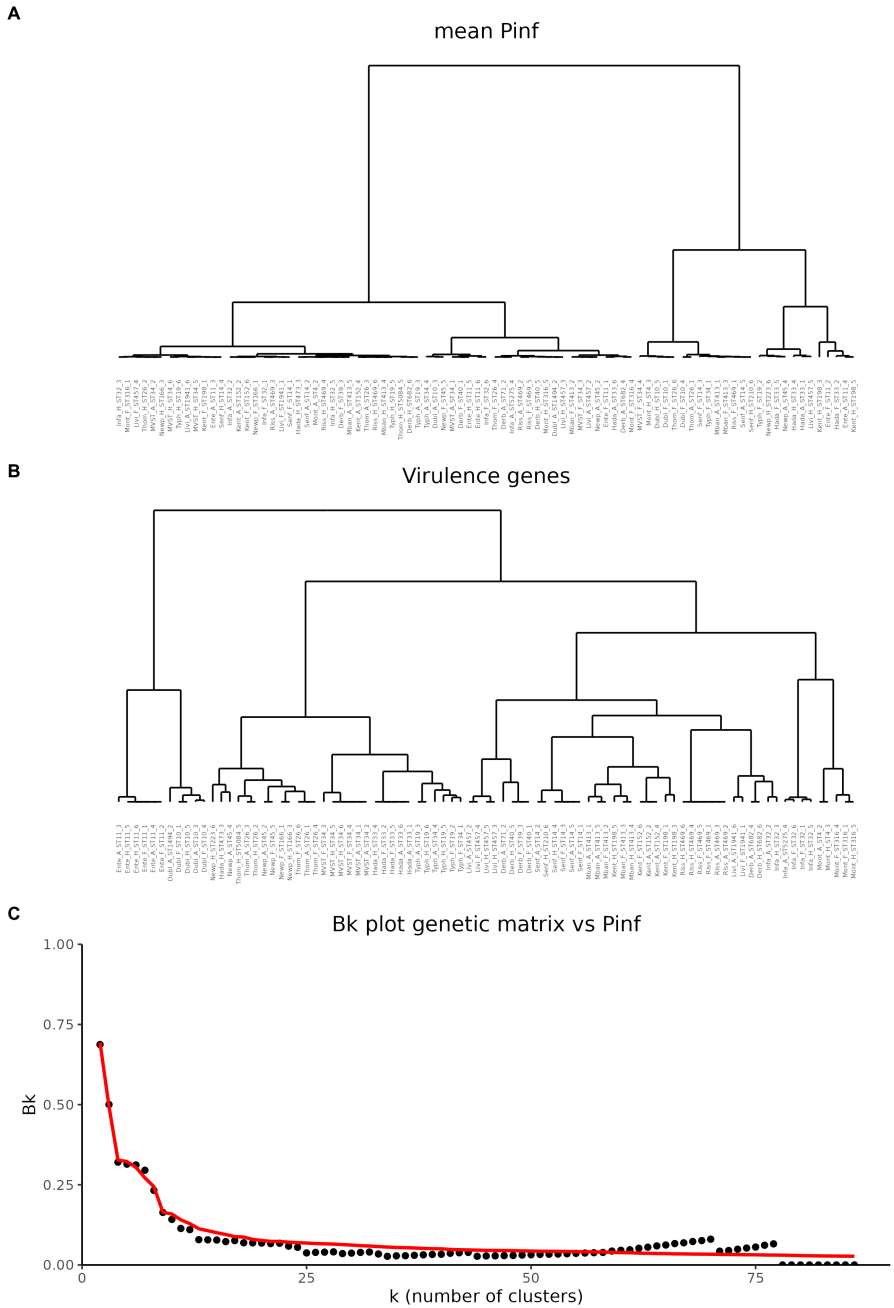
Comparison of hierarchical cluster trees. **(A)** Cluster tree based on the mean values of P(inf). **(B)** Cluster based on the matrix of virulence genes. **(C)** Comparison of the two cluster trees based on the B_k_ statistics. Black dots represent the B_k_ values plotted against the k number of clusters in which the dendrogram has been portioned. Red line represents the one-sided rejection region based on the asymptotic distribution of B_k_ for each value of k under the null hypothesis of no relation between the clusters.

To investigate potential associations between the mean value of P(inf) and the presence or absence of specific virulence genes, cluster trees were built for both the mean P(inf) of each isolate and the genes presence/absence. Cluster trees were then compared to quantify similarities. Figure 3 shows the cluster trees based on the mean value of P(inf) (Figure 3A) and based on the matrix of presence/absence of virulence genes (Figure 3B). Looking at the cluster trees, it was noticed that both the elements clustered together, and that the branching structures are different. In order to assess the similarity between the two clusters, the *B*_k_ statistic was computed and plotted as a function of the number of sub-clusters *k* in which the two trees could be partitioned into (black dots in Figure 3C). The red line in Figure 3C represents the 95% rejection region under the null hypothesis of no relation between the trees. For the majority of the *k* partitions, the black dots fall below the red line, indicating that the tree based on the P(inf) and the tree based on the virulence genetic matrix are not related.

### 3.4. Predicting mean P(inf) from virulence genes and ability to produce biofilm

**Figure 4:**
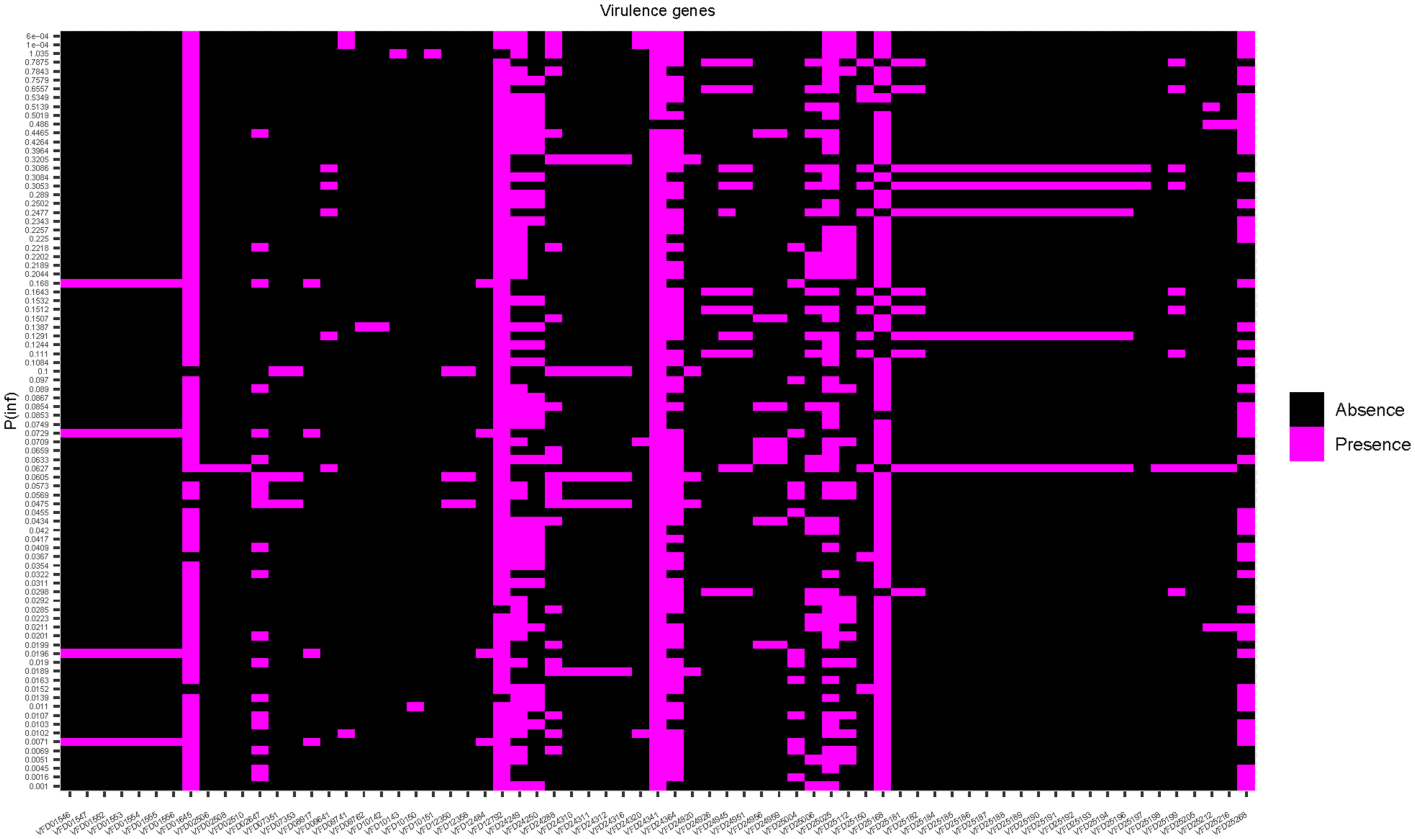
Heat map showing the presence/absence of genes for each isolate. Strains are ranked on the Y-axis according to the mean P(inf) values.

**Figure 5:**
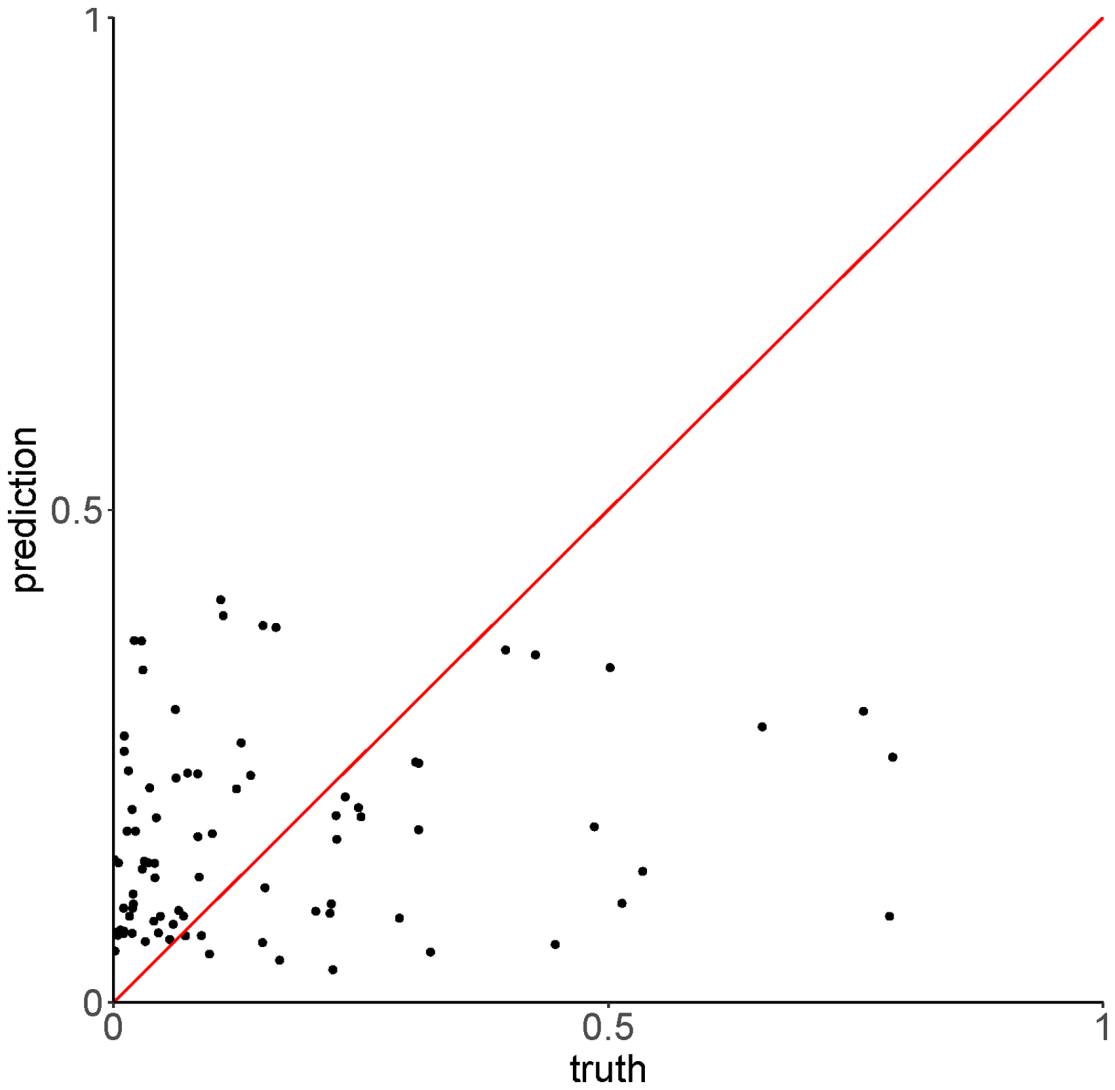
Out of bag (OOB) predictions of the Random Forest model run in regression mode versus the true values of P(Inf).

To assess whether the mean value of P(inf) could be predicted by the presence/absence of specific genes, a Random Forest (RF) model was applied. First, presence/absence of virulence genes was plotted against the mean values of P(inf) for each isolate (Figure 4). No evident pattern(s) were found to link the presence of a given virulence gene(s) and the mean P(inf) of the isolates. The percentage of variance explained for the RF model run in regression mode with 10.000 trees was essentially 0. This low score indicated that the virulence genes were very poor predictors of the mean value of P(inf). This was also confirmed by plotting the out of bag (OOB) predictions versus the true mean values of P(inf) (Figure 5).

The OOB error rate for the model run in classification mode with 10.000 trees (dichotomy variable “low,” “high”) was 42.5%. For both regression and classification models, increasing the number of trees did not improve the performance. The accuracy of the model in classification mode was 59%, with 95% CI [36–79%]. This showed that the presence/absence of virulence genes were poor predictors of the mean P(inf) also when treated as dichotomous variable.

The RF analysis was conducted also using the informative sequences, ability to form biofilm and serovar as predictors. Results are detailed in Supplementary material S1.

## 4. Discussion

For many years, salmonellosis has been the second most commonly reported foodborne bacterial infection in Europe (EFSA and ECDC, 2021a). EU Regulation (CE) No 2160/2003 and national control programs were implemented to lower the prevalence of *Salmonella* serovars relevant for public health, however, the choice of which serovars to be included as relevant in poultry flock has been the recent object of further evaluation, due to the observation that no further significant decrease in human salmonellosis infections has occurred since 2012 in EU (Koutsoumanis et al., 2019b). The large amount of available molecular and genomic data in association with epidemiological data, has allowed the identification of emerging *Salmonella* clones with a potentially high impact on human and public health. Indeed, the emergence of such high-risk clones, especially those characterized by increased virulence or resistance to relevant antimicrobials for human medicine, usually precedes the epidemic spread of a specific serovar, with the majority of the stains within such serovar not belonging to the emergent clone and displaying instead only limited epidemiological relevance (Aviv et al., 2014; Mourão et al., 2014; Worley et al., 2018; Chiou et al., 2019; Liao et al., 2019; Yokoyama et al., 2019). It would therefore be important to identify a panel of biological markers that could predict the relevance of a strain in terms of public health impact, besides the serovars.

In this study we determined the *in vitro* infectivity (probability of infection, or P(inf)), of a panel of *Salmonella* strains, investigated the presence of virulence genes in their genomes and integrated these data to identify potential predictors of P(inf), with the final aim of highlighting potentially relevant *Salmonella* strains for public health.

The use of P(inf) as a proxy of infectivity, and ultimately virulence, of a given strain has been driven by the fact that *Salmonella* can act as intracellular pathogen able to invade the intestinal mucosa through expression of genes encoded in the *Salmonella* Pathogenicity Island (SPI)-1 (Galán, 2001). Moreover, invasive NTS (iNTS), which spreads to host tissues beyond the intestine, can cause bacteremia and infection of usually sterile sites (Uzzau et al., 2000; Santos et al., 2009). Many serovars have been documented to cause iNTS, including *S. Typhimurium*, *S. Enteritidis* and *S*. Dublin (Uzzau et al., 2000; Okoro et al., 2015; Feasey et al., 2016; Mughini-Gras et al., 2020). Although these serovars mainly cause self-limiting gastroenteritis, their potential to cause more severe, invasive infections should not be underestimated. For this reason, P(inf), as derived from the INV to ON ratio, has been considered as an ultimate indicator of infectivity providing a snapshot of the most severe form of *Salmonella* infection.

The set of strains showed a wide range of P(inf) values ranging from 10^−5^ to 10^−1^, similar to what has been reported for another *Salmonella* strain collection by Kuijpers et al. (2019), thus indicating variability among serovars and individual strains within a serovar. This is not surprising, since the large variability expressed by *Salmonella* serovars and strains in terms of virulence mechanisms (Cheng et al., 2019) could influence their ability to invade epithelial human cells. Indeed, our results suggested that generally P(inf) for food *Salmonella* isolates of a given serovar was higher than P(inf) for animal and human strains within the same serovar., with the exception of *S*. Kentucky, *S*. Livingstone and *S*. Montevideo, where P(inf) of human strains showed higher values. These serovars are uncommon in human infections, accounting for 0.69, 0.25 and 0.31% of the confirmed reported human cases in Europe in 2019, respectively (EFSA and ECDC, 2021b; European Centre for Disease Prevention and Control (ECDC), 2022).

It is interesting to note that in this study, *S*. Kentucky sequence type (ST) 152 strains, usually associated to poultry (Haley et al., 2016; Hawkey et al., 2019), displayed lower P(inf) values than *S*. Kentucky strains ST198, usually associated with humans (le Hello et al., 2013; Hawkey et al., 2019). Indeed, Le Hello et al. (2011) reported that patients infected with ciprofloxacin-resistant (CIP^R^) *S*. Kentucky ST198 strains, a clone that rapidly spread in recent years due to the emergence of CIP^R^ and multidrug resistance (Coipan et al., 2020), were more frequently hospitalized, possibly reflecting the higher invasive potential of this clone. In a different study, using an *in vitro* assays with chicken embryo hepatocytes, *S*. Kentucky strains isolated from poultry sources exhibited equal invasive capabilities to that of other serovars, including *S. enteritidis* and *S. Typhimurium*, while they resulted in less invasion than isolates of *S. Enteritidis*, *S*. Mbandaka, and *S. Typhimurium* in invasion assays with a human ileocecal adenocarcinoma cells line (HCT-8 cells) (Joerger et al., 2009). On the basis of its rapid emergence and dissemination in poultry and humans, France has included *S*. Kentucky as a target serovar in poultry flocks (Koutsoumanis et al., 2019b). It is therefore possible that the ability of *S*. Kentucky strains to invade cells depends on the cell line used and also on sequence type, even if the reasons why certain *S*. Kentucky STs are associated with different hosts are not completely elucidated.

Human cases of salmonellosis caused by *S*. Montevideo are also infrequent, however this serovar has been described as the causative agent in cases of bacteremia (Kim et al., 2004), serous and septic arthritis (Gordon et al., 1949; Katsoulis et al., 2004), and acute myocarditis (O’Connor, 2000), demonstrating its potential as invasive pathogen in human infections. Foodborne outbreaks associated to this serovar have also been documented, with pepper, sesame seeds and their products listed as food vehicles (Unicomb et al., 2005; Willis et al., 2009; D’Oca et al., 2021), and low concentrations of *S*. Montevideo were found sufficient in such products to cause outbreaks (Unicomb et al., 2005; Harada et al., 2011; Stöcker et al., 2011; Paine et al., 2014). In the current study, indeed the two *S*. Montevideo isolates from human sources, despite having different P(inf) values, were among the human strains with the highest ability to invade Caco-2 cells, and showed higher P(inf) values compared to *S. typhimurium*, MVST and *S. Enteritidis* strains isolated from human sources, although such serovars are frequently found in human infections. Furthermore, Lalsiamthara and Lee (2017) demonstrated that the competence of *S*. Montevideo strains in invading chicken epithelial cells is comparable to that of *S. Typhimurium*, with whom *S*. Montevideo shares similar pattern of macrophage uptake and survival (Lalsiamthara and Lee, 2017).

Other serovars often involved in human infections, for which we expected higher ability to invade human cells, on the contrary, showed a higher variability in P(inf) values, and we even observed that the strains isolated from human cases showed lower P(inf) values than strains isolated from animal and food sources.

*Salmonella Typhimurium* and MVST, for instance have been consistently reported as major cause of human salmonellosis in Europe and worldwide (Ferrari et al., 2019; EFSA and ECDC, 2021b). Consumption of contaminated pork and poultry products was identified as the primary source of infection (EFSA and ECDC, 2021a), even if other food sources have been linked to outbreaks recently (Larkin et al., 2022; Lund et al., 2022). The higher P(inf) values displayed by strains isolated from animal and food sources could suggest that the strains isolated from non-human sources are in fact more efficient in invading human cells and possibly causing an infection in humans. Further studies are needed to understand why, but the observation may give hints, why human-to-human spread of non-typhoidal *Salmonella* is known to be relatively rare. For instance, Kapperud et al. (1990) demonstrated that infection dose for *S. Typhimurium* in a Norwegian chocolate outbreak was very low in the food vehicle, probably due to the high level of fat, which protects the pathogen from gastric acidity through the stomach. Of note, the recent *Salmonella* outbreak related to chocolate products in Europe, which was characterized by a hospitalization rate of about 40% and some cases with severe clinical symptoms, was caused by MVST ST34, which in the current study also displayed a high P(inf). Similarly the different ability to invade Caco-2 cells in *S.* Kentucky according to ST, *S. Typhimurium* ST19 isolates generally displayed lower P(inf) values compared to *S. Typhimurium* ST34 isolates. Nonetheless, food isolates, irrespectively of the ST, had the highest P(inf). Variability in P(inf) values was also observed in *S. Enteritidis* and *S. Infantis* strains, where isolates from human sources were less invasive than isolates from animal and food sources, despite they were all of ST11 and ST32, respectively. Further research is needed to elucidate the variability within these serovars, since they are often involved in human infections (EFSA and ECDC, 2017, 2021a,b; Sarno et al., 2021).

High values of P(inf) were shown for *S*. Hadar isolates from food sources. Although this serovar is rarely reported from human infections, and caused only 298 human infections in 2019 in Europe (0.41% of the reported cases, European Centre for Disease Prevention and Control (ECDC), 2022), it is included as a target serovar in national programs for *Salmonella* control in breeding flocks of *Gallus gallus*. This serovar has been recently considered for revision due to its low frequency in breeders, broilers, layer flocks and humans; however, our data indicate that *S*. Hadar strains, irrespectively of their origin or ST, are able to efficiently pass through the *in vitro* GIT system and invade human cells, potentially developing an infection. Similar considerations can be drawn for *S*. Dublin isolates that in the current study showed high ability to invade human cells: indeed, human infections with this serovar often present as fatal syndromes (Fang and Fierer, 1991; Helms et al., 2003) and as reported by other researchers, *S*. Dublin isolates showed higher invasiveness and pathogenicity on Caco-2 cells (Bolton et al., 2000; Mughini-Gras et al., 2020).

For the other serovars included in the current study, similar scenarios can be described: *S*. Mbandaka, *S*. Rissen, *S*. Senftenberg and *S.* Thompson displayed variability in their ability to invade human cells after the passages in the GIT system, despite belonging to the same STs. On the contrary, *S*. Livingstone and *S*. Newport, for which we included isolates of different STs, showed similar P(inf) values, while for *S*. Derby we could describe diversity in the ability to invade human cells in the GIT system but no relation to the different STs characterizing our *S*. Derby strains and the circulating clones (Sévellec et al., 2020).

Overall, we did not find statistically significant differences among serovars in the different passages of the GIT system, except for adhesiveness to Caco-2 cells. Adhesion to surfaces and host cells is mediated by fimbriae that are therefore recognized as important virulence factors: since different fimbrial gene clusters exist in *Salmonella* spp. and most of them are sporadic or found only in few strains (Dufresne and Daigle, 2017). It is therefore possible that the different prevalence of certain fimbriae among *Salmonella* serovars influence their ability to adhere to Caco-2 cells. Nonetheless, the higher adhesiveness shown by *S. Typhimurium* strains did not always result in a better ability to invade cells and probably other factors involved in the invasion mechanisms could explain these differences.

The association of P(inf) with biofilm formation ability was also studied and statistically significant associations were found in all the different conditions for a limited number of serovars (*S. Infantis*, *S*. Hadar, *S. Enteritidis*, *S*. Derby, *S*. Montevideo), indicating that the two phenotypes are governed by different mechanisms and for the majority of the serovars tested here the ability to form biofilm does not seem to correlate with the ability to invade epithelial cells. Indeed, it seems that for *S*. Montevideo, *S. Infantis* and *S*. Derby strains, biofilm formation especially if the strains are exposed at pH 4.5, although limited (Petrin et al., 2022), impairs significantly the ability of such strains to invade human Caco-2 cells. On the contrary, from our data, a positive correlation between biofilm formation and P(inf) emerged for *S*. Hadar and *S. Enteritidis* strains. Previous studies demonstrated that high-virulence *S. Enteritidis* strains, incubated at optimal biofilm-forming conditions, release a soluble factor enabling them to disrupt the integrity of Caco-2 monolayer (Solano et al., 2001, 2002), while others found no correlation and suggested that biofilm formation might enhance cell invasiveness, even if this trait is not essential for cell invasiveness of *S. Enteritidis* in cultured epithelial cells (Shah et al., 2011). Most of the *S*. Hadar and few *S. Enteritidis* strains indeed showed high ability to invade human Caco-2 cell in the current study. For all the other tested serovars, no significant correlation was found, supporting the hypothesis that different characteristics other than the ability to form biofilm contribute to *Salmonella* invasiveness.

We then tried to predict the P(inf) from the presence or absence of virulence genes, however, the Random Forest model did not show good performance. It might then be that the differences in P(inf) are not determined by presence or absence of genes, but rather by difference in expression of genes present in all serovars. Similarly, the informative sequences, present only in at least 10% and no more than 95% of the genomes, as well as biofilm formation ability or serovar were only poor predictors of P(inf).

Although the well-differentiated Caco-2 cells closely mimic the differentiated intestinal tract (Hara et al., 1993) and previous studies have used them alone to successfully identify how *Salmonella* invasiveness differs (Solano et al., 2001; Betancor et al., 2009; Yim et al., 2010) or in an *in vitro* GIT system to identify genes potentially associated with P(inf) (Kuijpers et al., 2019), our study did not lead to conclusive results on the identification of specific virulence genes to predict the pathogenicity of a given *Salmonella* strain. Indeed, other factors might be involved in the infection process and these factors may depend on the pathogen itself, but also on other elements, such as the host environment, which is usually complex and cannot be properly reproduced in an *in vitro* model. Indeed, as already discussed by Wijnands et al. (2017), the GIT model used in our study does not take into account, for example the mechanical movements enabling the chyme to pass through the gastrointestinal tract, nor the role of immune system and intestinal microbiota, which can play an essential role in influencing *Salmonella* survival, attachment and invasion abilities.

Different approaches, such as RNAseq or metabolic profiling, together with more complex *in vitro* systems, could be helpful to understand further the genetic characteristics involved in the invasiveness ability of each *Salmonella* strain.

## 5. Conclusion

We assessed the virulence of a set of *Salmonella* strains using an *in vitro* gastrointestinal tract model system that also includes attachment to and invasion of cultured human Caco-2 cells. The probability of infection P(inf) was estimated as a quantification of the infectivity of each strain and statistical associations with the ability of such strains to produce biofilm and the presence or absence of virulence genes were studied. Large variability in P(inf) was observed between *Salmonella* strains, and serovars more commonly isolated from human infections did not always show greater P(inf) nor invasiveness. Moreover, it was not possible to identify virulence genes that acted as predictors of P(inf) unambiguously. The use of expression data and/or metabolic profiles could represent a valuable alternative to detect genes highly associated with P(inf) and could be used to test potential association with strain’s invasiveness.

## Data availability statement

The datasets generated and analyzed during the current study are available in the NCBI database (http://www.ncbi.nlm.nih.gov) under BioProject ID PRJNA817603.

## Author contributions

SP performed the experiments and wrote the manuscript. LW and EHMD-vA performed the experiments. EB, LM-G, and MO performed the analyses. CL, LB, and JO contributed to the concept of the work. All authors contributed to the article and approved the submitted version.

## Funding

This work was supported by the project ‘PRoSPECT’: Predicting *Salmonella* Pathogenic Potential to Enhance Targeted Control Strategies’, funded by the Italian Ministry of Health (grant no. RF-2018-12366604).

## Conflict of interest

The authors declare that the research was conducted in the absence of any commercial or financial relationships that could be construed as a potential conflict of interest.

## Publisher’s note

All claims expressed in this article are solely those of the authors and do not necessarily represent those of their affiliated organizations, or those of the publisher, the editors and the reviewers. Any product that may be evaluated in this article, or claim that may be made by its manufacturer, is not guaranteed or endorsed by the publisher.

## Footnotes

^1^ www.enterobase.warwick.ac.uk
