# Supplementary material for: Assessing phenotypic virulence of Salmonella enterica across serovars and sources
Source: Front Microbiol. 2023 Jun 6;14:1184387. doi: 10.3389/fmicb.2023.1184387 (PMC10279978; doi:10.3389/fmicb.2023.1184387)
Supplement: Supplementary file 1 [file Data_Sheet_1.zip › Table 2 - 2023-05-15T095156.952.DOCX]

**Supplementary Table S2.** Summary of results from the gastro-intestinal tract (GIT) model system assay of the 87 *Salmonella* isolates. A = animal, F = food, H = human sources.

| ***Salmonella* strain ID** | **Serovar** | **Source** | **Isolation year** | **P(inf)** | ***Salmonella* strain ID** | **Serovar** | **Source** | **Isolation year** | **P(inf)** |
| --- | --- | --- | --- | --- | --- | --- | --- | --- | --- |
| **Derb_A_ST682_17-43403** | Derby | A | 2013 | 4,7E-02 | **Mban_F_ST413_19-96061** | Mbandaka | F | 2018 | 3,1E-02 |
| **Derb_A_ST71_17-94877** | Derby | A | 2013 | 3,2E-02 | **Mban_H_ST413_Villa-11-71-37-14** | Mbandaka | H | 2014 | 5,8E-03 |
| **Derb_F_ST39_17-43426** | Derby | F | 2012 | 1,2E-02 | **Mont_A_ST4_19-109691** | Montevideo | A | 2016 | 1,4E-02 |
| **Derb_F_ST40_17-43372** | Derby | F | 2009 | 8,8E-02 | **Mont_F_ST316_19-123375** | Montevideo | F | 2010 | 5,6E-02 |
| **Derb_H_ST40_17-94894** | Derby | H | 2014 | 1,9E-02 | **Mont_F_ST316_19-123380** | Montevideo | F | 2010 | 2,6E-02 |
| **Derb_H_ST682_17-106726** | Derby | H | 2011 | 4,6E-03 | **Mont_H_ST316_Villa-14-13-17-19** | Montevideo | H | 2018 | 3,2E-02 |
| **Dubl_A_ST10_17-141144** | Dublin | A | 2017 | 8,1E-02 | **Mont_H_ST4_20-27307** | Montevideo | H | 2018 | 1,6E-01 |
| **Dubl_A_ST1494_17-141138** | Dublin | A | 2017 | 1,9E-02 | **MVST_A_ST34_17-71879** | MVST | A | 2016 | 8,6E-02 |
| **Dubl_F_ST10_19-10947** | Dublin | F | 2016 | 1,8E-01 | **MVST_A_ST34_17-71883** | MVST | A | 2016 | 2,2E-02 |
| **Dubl_F_ST10_19-109645** | Dublin | F | 2019 | 9,4E-02 | **MVST_F_ST34_17-71864** | MVST | F | 2016 | 4,2E-02 |
| **Dubl_H_ST10_20-77715** | Dublin | H | 2014 | 1,4E-01 | **MVST_F_ST34_17-71882** | MVST | F | 2016 | 1,5E-01 |
| **Ente_A_ST11_16-80468** | Enteritidis | A | 2016 | 1,7E-02 | **MVST_H_ST34_20-27314** | MVST | H | 2018 | 1,8E-02 |
| **Ente_A_ST11_18-26234-72** | Enteritidis | A | 2015 | 4,0E-01 | **MVST_H_ST34_Lucarelli-22-16-09-18_S22** | MVST | H | 2018 | 1,6E-02 |
| **Ente_F_ST11_19-109519** | Enteritidis | F | 2017 | 3,9E-01 | **Newp_A_ST45_19-109588** | Newport | A | 2017 | 1,7E-01 |
| **Ente_F_ST11_19-109561** | Enteritidis | F | 2017 | 5,3E-02 | **Newp_A_ST45_19-109590** | Newport | A | 2017 | 4,4E-02 |
| **Ente_H_ST11_16-88911** | Enteritidis | H | 2016 | 9,4E-02 | **Newp_F_ST166_19-96037** | Newport | F | 2018 | 5,8E-04 |
| **Ente_H_ST11_Lucarelli-16-19-14-19_S16** | Enteritidis | H | 2017 | 6,9E-02 | **Newp_F_ST45_19-98047** | Newport | F | 2018 | 1,1E-01 |
| **Hada_A_ST33_19-123384** | Hadar | A | 2011 | 6,1E-02 | **Newp_H_ST166_Villa-05-38-5-17** | Newport | H | 2016 | 2,3E-02 |
| **Hada_A_ST33_19-123385** | Hadar | A | 2011 | 2,3E-01 | **Newp_H_ST223_Villa-09-37-13-19** | Newport | H | 2016 | 2,1E-01 |
| **Hada_F_ST33_19-112221** | Hadar | F | 2013 | 2,5E-01 | **Riss_A_ST469_19-109581** | Rissen | A | 2017 | 6,2E-03 |
| **Hada_F_ST33_19-96040** | Hadar | F | 2018 | 3,8E-01 | **Riss_A_ST469_19-112167** | Rissen | A | 2014 | 2,7E-02 |
| **Hada_H_ST33_20-77714** | Hadar | H | 2018 | 2,3E-01 | **Riss_F_ST469_19-95995** | Rissen | F | 2018 | 1,2E-01 |
| **Hada_H_ST473_20-77712** | Hadar | H | 2019 | 5,3E-03 | **Riss_F_ST469_19-98836** | Rissen | F | 2018 | 1,8E-02 |
| **Infa_A_ST32_18-97049-67** | Infantis | A | 2017 | 8,3E-04 | **Riss_H_ST469_20-27313** | Rissen | H | 2018 | 8,9E-03 |
| **Infa_A_ST5275_18-97049-43** | Infantis | A | 2015 | 3,0E-02 | **Riss_H_ST469_Villa-31-68-29-18** | Rissen | H | 2018 | 6,4E-03 |
| **Infa_F_ST32_18-97049-46** | Infantis | F | 2015 | 9,5E-02 | **Senf_A_ST14_19-109705** | Senftenberg | A | 2016 | 6,5E-03 |
| **Infa_F_ST32_19-96052** | Infantis | F | 2018 | 3,6E-03 | **Senf_A_ST14_19-98049** | Senftenberg | A | 2018 | 1,0E-01 |
| **Infa_H_ST32_20-27302** | Infantis | H | 2017 | 1,0E-02 | **Senf_F_ST14_19-109603** | Senftenberg | F | 2017 | 2,7E-03 |
| **Infa_H_ST32_Lucarelli-19-55-20-18_S19** | Infantis | H | 2018 | 2,1E-02 | **Senf_F_ST14_19-112188** | Senftenberg | F | 2014 | 1,0E-01 |
| **Kent_A_ST152_19-96053** | Kentucky | A | 2018 | 6,7E-05 | **Senf_H_ST14_Villa-27-47-14-18** | Senftenberg | H | 2019 | 1,7E-02 |
| **Kent_A_ST152_19-98035** | Kentucky | A | 2018 | 3,6E-03 | **Senf_H_ST210_Villa-17-77-14-18** | Senftenberg | H | 2018 | 6,1E-02 |
| **Kent_F_ST152_19-96025** | Kentucky | F | 2018 | 3,1E-04 | **Thom_A_ST26_19-109613** | Thompson | A | 2017 | 2,0E-01 |
| **Kent_F_ST198_19-98826** | Kentucky | F | 2018 | 1,6E-02 | **Thom_A_ST26_19-96066** | Thompson | A | 2018 | 5,2E-03 |
| **Kent_H_ST198_20-27309** | Kentucky | H | 2019 | 4,3E-01 | **Thom_F_ST26_19-109609** | Thompson | F | 2017 | 4,1E-02 |
| **Kent_H_ST198_Lucarelli-26-23-02-18** | Kentucky | H | 2015 | 5,2E-01 | **Thom_F_ST26_19-123367** | Thompson | F | 2011 | 1,8E-01 |
| **Livi_A_ST1941_19-109673** | Livingstone | A | 2016 | 1,7E-02 | **Thom_H_ST26_Villa-25-53-11-16** | Thompson | H | 2016 | 2,5E-02 |
| **Livi_A_ST457_19-38110-18** | Livingstone | A | 2018 | 5,3E-02 | **Thom_H_ST5084_20-27315** | Thompson | H | 2018 | 7,2E-03 |
| **Livi_F_ST1941_19-109566** | Livingstone | F | 2017 | 1,2E-03 | **Typh_A_ST19_19-96016** | Typhimurium | A | 2018 | 1,0E-02 |
| **Livi_F_ST457_19-96011** | Livingstone | F | 2018 | 2,9E-02 | **Typh_A_ST34_699-119** | Typhimurium | A | 2017 | 5,8E-02 |
| **Livi_H_ST457_20-27308** | Livingstone | H | 2019 | 1,8E-01 | **Typh_F_ST19_19-96065** | Typhimurium | F | 2018 | 2,8E-01 |
| **Livi_H_ST457_Villa-22-88-15-19** | Livingstone | H | 2017 | 3,3E-02 | **Typh_F_ST34_19-98051** | Typhimurium | F | 2018 | 1,3E-01 |
| **Mban_A_ST413_19-109690** | Mbandaka | A | 2016 | 1,1E-02 | **Typh_H_ST19_20-77702** | Typhimurium | H | 2016 | 5,0E-03 |
| **Mban_A_ST413_19-98037** | Mbandaka | A | 2018 | 1,1E-01 | **Typh_H_ST19_20-77710** | Typhimurium | H | 2016 | 2,0E-02 |
| **Mban_F_ST413_19-109574** | Mbandaka | F | 2017 | 1,1E-01 |  |  |  |  |  |
