# Supplementary material for: Assessing phenotypic virulence of Salmonella enterica across serovars and sources
Source: Front Microbiol. 2023 Jun 6;14:1184387. doi: 10.3389/fmicb.2023.1184387 (PMC10279978; doi:10.3389/fmicb.2023.1184387)
Supplement: Supplementary file 1 [file Data_Sheet_1.zip › Table 1 (28).docx]

**Supplementary Material and Methods and Results for ‘Assessing the phenotypic virulence of *Salmonella enterica* across serovars and sources’**

**Supplementary Material and Methods**

1. **Simulated gastric fluid (SGF) composition and preparation**

The composition of the simulated gastric fluid (SGF) is based on the study by Rotard and colleague (1995) and the preparation as by Oliveira et al. (2011). More specifically, SGF is composed of NaCl (175.0 g/L), NaH_2_PO_4_ (88.8 g/L), KCl (89.6 g/L), CaCl_2_ (22.2 g/L), NH_4_Cl (30.6 g/L), D+-glucose (65.0 g/L), D-glucuronic-acid (2.0 g/L), urea (25.0 g/L), glucosamine (33.0 g/L), bovine serum albumin fraction V (1.0 g/L), type II mucin from porcine stomach (3.0 g/L) and pepsin (1.3 g/L). The solution was filter sterilized and after that, radiation-sterilized mucin and pepsin were added. The final SGF solution was mixed overnight at room temperature and pH was set at 2.5±0.1 before use.

1. **Simulated intestinal fluid (SIF) composition and preparation**

SIF solution consists of two solutions, basic intestinal solution and bile solution. The former is composed of NaCl (175.3 g/L), NaHCO_3_ (84.7 g/L), KH_2_PO_4_ (8.0 g/L), KCl (89.6 g/L), MgCl_2_ (5.0 g/L), urea (25.0 g/L), CaCl_2_ (29.8 g/L), bovine serum albumin fraction V (1.0 g/L), lipase (0.5 g/L) and pancreatin (3.0 g/L). The solution was filter sterilized and after that, radiation-sterilized lipase and pancreatin were added. pH was set at 7.8±0.2 and the basic SIF solution was mixed overnight at room temperature. The bile solution is composed of NaCl (175.3 g/L), NaHCO_3_ (84.7 g/L), KCl (89.6 g/L), urea (25.0 g/L), CaCl_2_ (29.8 g/L), bovine serum albumin fraction V (1.8 g/L), and bile (6.0 g/L). Radiation-sterilized bile was added after filter sterilization of the solution. pH was set at 8.0±0.2 and the bile solution was mixed overnight at room temperature. Three parts of SIF basic solution and one part of bile solution were mixed to obtain the final SIF solution.

**Supplementary Results**

To assess whether the mean value of P(inf) could be predicted by the presence/absence of informative sequences, ability to form biofilm and serovar, a Random Forest (RF) model was applied. The presence/absence of predictors was plotted against the mean values of P(inf) for each isolate (**Figure S1**). No evident pattern(s) were found to link the presence of informative sequences, biofilm formation and serovar and the mean P(inf) of the isolates. The percentage of variance explained for the RF model run in regression mode with 10.000 trees was 3.7%. This low score indicated that the features used in the model were very poor predictors of the mean value of P(inf). This was also confirmed by plotting the out of bag (OOB) predictions versus the true mean values of P(inf) (**Figure S2**). The OOB error rate for the model run in classification mode with 10.000 trees (dichotomous variable “low” and ”high”) was 42.5%. For both regression and classification models, increasing the number of trees did not improve the performance. The accuracy of the model in classification mode was 59%, with 95% CI [36%-79%]. This showed that the features used in the model were very poor predictors of the mean P(inf) also when treated as dichotomous variables.


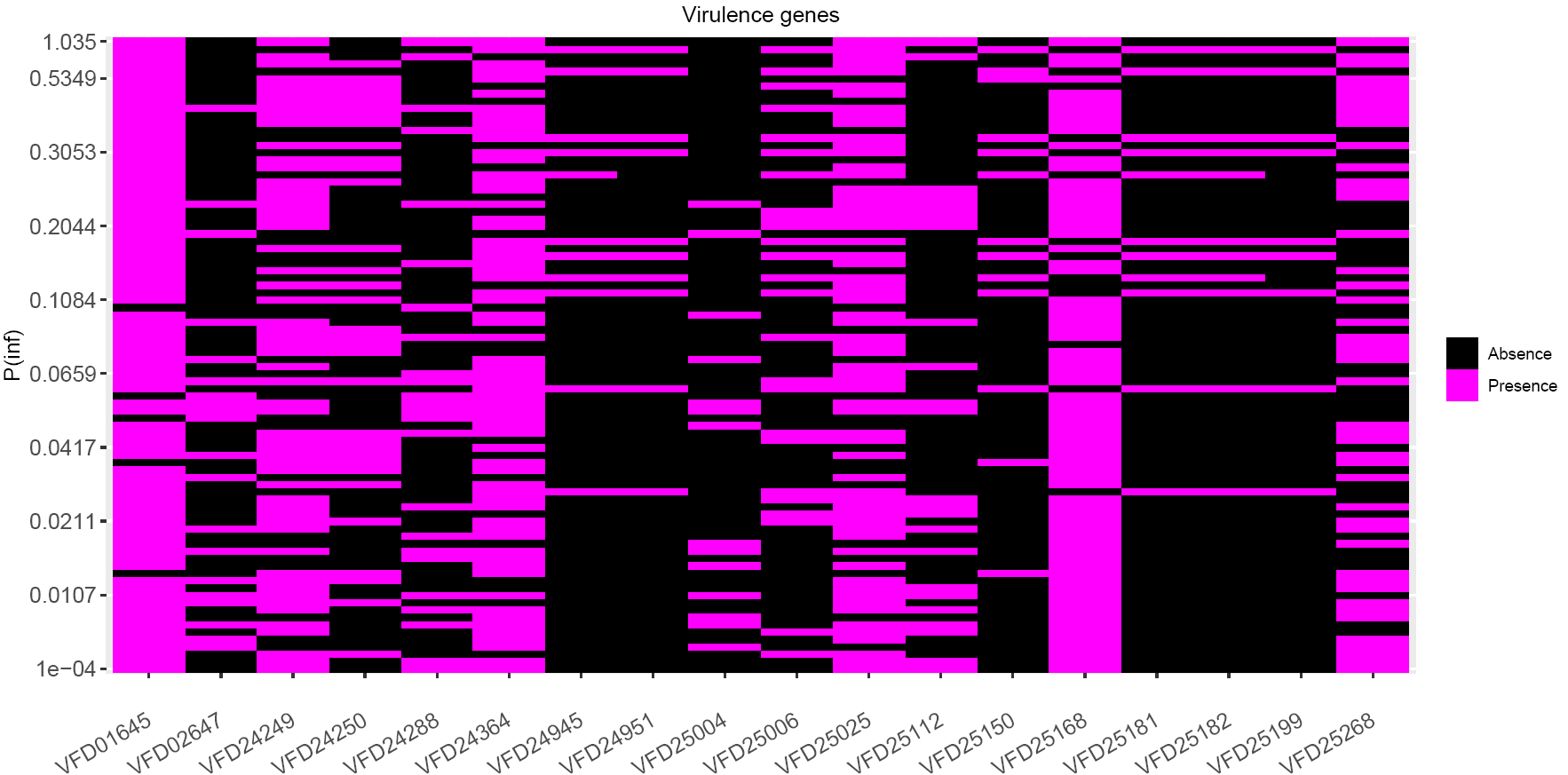


**Figure S1**. Heat map showing the presence/absence of informative genes for each isolate. Strains are ranked on the Y-axis according to the mean P(inf) values.


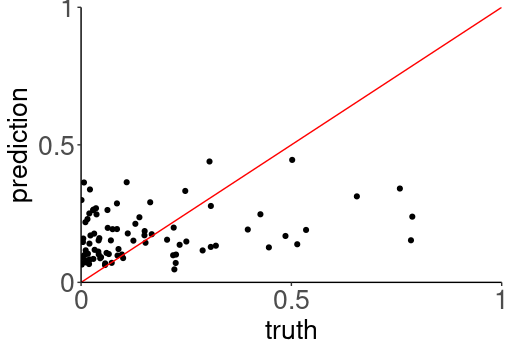


**Figure S2.** Out of bag (OOB) predictions of the Random Forest model run in regression mode versus the true values of P(Inf).

1. **References**

Oliveira, Marcia, Lucas Wijnands, Maribel Abadias, Henk Aarts, and Eelco Franz. 2011. “Pathogenic Potential of Salmonella Typhimurium DT104 Following Sequential Passage through Soil, Packaged Fresh-Cut Lettuce and a Model Gastrointestinal Tract.” *International Journal of Food Microbiology* 148 (3): 149–55. https://doi.org/10.1016/J.IJFOODMICRO.2011.05.013.

Rotard, Wolfgang, Wilfried Christmann, Wilhelm Knoth, and Wolfgang Mailahn. 1995. “Bestimmung Der Resorptionsverfügbaren PCDD/PCDF Aus Kieselrot - Simulation Der Digestion Mit Böden.” *Umweltwissenschaften Und Schadstoff-Forschung* 7 (1): 3–9. https://doi.org/10.1007/BF02938733/METRICS.
